# Assessment of survival in patients with idiopathic pulmonary fibrosis using quantitative HRCT indexes

**DOI:** 10.1186/s40248-018-0155-2

**Published:** 2018-12-01

**Authors:** Sebastiano Emanuele Torrisi, Stefano Palmucci, Alessandro Stefano, Giorgio Russo, Alfredo Gaetano Torcitto, Daniele Falsaperla, Mauro Gioè, Mauro Pavone, Ada Vancheri, Gianluca Sambataro, Domenico Sambataro, Letizia Antonella Mauro, Emanuele Grassedonio, Antonio Basile, Carlo Vancheri

**Affiliations:** 1 Regional Referral Centre for Rare Lung Diseases, A.O.U. Policlinico-Vittorio Emanuele, Department of Clinical and Experimental Medicine, University of Catania, via Santa Sofia 78, Catania, Italy; 2 Radiology I Unit, Department of Medical Surgical Sciences and Advanced Technologies, University Hospital “Policlinico-Vittorio Emanuele”, Catania, Italy; 3 National Research Council (IBFM-CNR), Contrada Pietropollastra-Pisciotta, Institute of Molecular Bioimaging and Physiology, 90015 Cefalù, Italy; 4 Department of Brain and Behavioral Sciences, University of Pavia, Pavia, Italy; 5 Artroreuma srl, Outpatient of Rheumatology Accredited with National Health System, Corso San Vito 53, 95030 Mascalucia, CT Italy; 6 Section of Radiological Sciences, DIBIMEF, University Hospital “Paolo Giaccone”, University of Palermo, Palermo, Italy

**Keywords:** Idiopathic pulmonary fibrosis, HRCT, Kurtosis, Usual interstitial pneumonia, Survival, Mortality

## Abstract

**Background:**

The assessment of Idiopathic Pulmonary Fibrosis (IPF) using HRCT requires great experience and is limited by a significant inter-observer variability, even between trained radiologists. The evaluation of HRCT through automated quantitative analysis may hopefully solve this problem. The accuracy of CT-histogram derived indexes in the assessment of survival in IPF patients has been poorly studied.

**Methods:**

Forty-two patients with a diagnosis of IPF and a follow up time of 3 years were retrospectively collected; HRCT and Pulmonary Function Tests (PFTs) performed at diagnosis time were analysed; the extent of fibrotic disease was quantified on HRCT using kurtosis, skewness, Mean Lung Density (MLD), High attenuation areas (HAA%) and Fibrotic Areas (FA%). Univariate Cox regression was performed to assess hazard ratios for the explored variables and a multivariate model considering skewness, FVC, DL_CO_ and age was created to test their prognostic value in assessing survival. Through ROC analysis, threshold values demonstrating the best sensitivity and specificity in predicting mortality were identified. They were used as cut-off points to graph Kaplan-Meier curves specific for the CT-indexes.

**Results:**

Kurtosis, skewness, MLD, HAA% and FA% were good predictors of mortality (HR 0.44, 0.74, 1.01, 1.12, 1.06; *p* = 0.03, *p* = 0.01, *p* = 0.02, *p* = 0.02 and *p* = 0.017 respectively). Skewness demonstrated the lowest Akaike’s information criterion value (55.52), proving to be the best CT variable for prediction of mortality. Significant survival differences considering proposed cut-off points were also demonstrated according to kurtosis (*p* = 0.02), skewness (*p* = 0.005), MLD (*p* = 0.003), HAA% (*p* = 0.009) and FA% (*p* = 0.02) – obtained from quantitative HRCT analysis at diagnosis time.

**Conclusions:**

CT-histogram derived indexes may provide an accurate estimation of survival in IPF patients. They demonstrate a correlation with PFTs, highlighting their possible use in clinical practice.

## Background

Idiopathic pulmonary fibrosis (IPF) is a rare pulmonary disease characterized by a progressive fibrosis of the lungs that leads to the inexorable worsening of lung function [1]. IPF is also characterized by a very poor survival rate, ranging from 3 to 5 years from diagnosis, which is worse than in many cancers [2–5].

ILD assessment using HRCT requires great experience and is limited by a significant inter-observer variability, even between trained radiologists [6]. In the longitudinal evaluation of disease progression, some studies are based on a “visual” semi-quantitative analysis of HRCT; however, this method has been considered “tedious and not reproducible” [7].

A more objective evaluation of HRCT through automated quantitative analysis may hopefully solve this problem. Recent studies started exploring this second possibility using, in most cases, complex algorithms, able to perform texture analysis. However, although very promising, texture analysis requires high computational steps, currently reserving its use for a research setting. To date, only a few studies have focused on CT-histogram evaluation, which is a different quantitative analysis method requiring fewer computational steps than texture analysis. These characteristics of objectivity and relative simplicity make this analysis potentially usable and reproducible in a realistic clinical setting.

Therefore, the aim of this study is to evaluate in a real-life work setting, the prognostic value of histogram-based quantitative HRCT variables in predicting mortality in a cohort of patients affected by IPF.

## Methods

### Patients

**Fig. 1:**
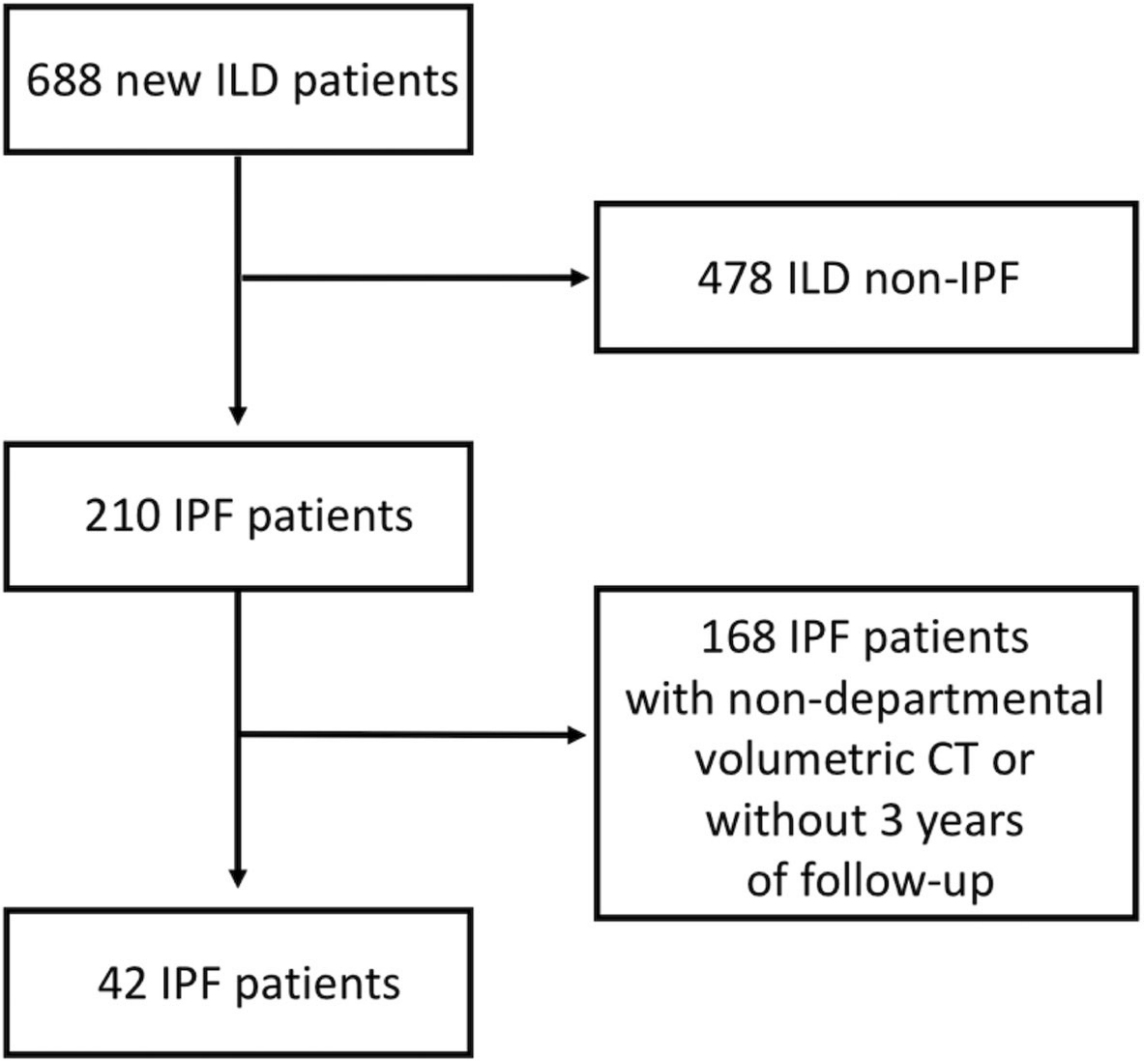
Flowchart reporting the selection process

A retrospective analysis of our interstitial lung disease (ILD) database identified a total of 210 patients who had received a multidisciplinary team (MDT) diagnosis of IPF according to 2011 ATS/ERS/JRS/ALAT IPF guidelines over a period of three years (January 2012 to August 2015) [1]. Only patients with an unenhanced, supine, volumetric thin-section CT exam performed in our departmental CT-scanner a well conducted clinical and functional follow up were included in the analysis. A median follow up time of 3 years was also required to assess survival. A total of 42 consecutive IPF patients fulfilling these criteria were finally included in the analysis (Fig. 1).

This study received approval the ethical committee of Policlinico-Vittorio Emanuele Hospital of Catania.

### Clinical information

All clinical information was obtained from medical records. PFTs (Forced Vital Capacity (FVC) and Diffusing Capacity for carbon monoxide (DL_CO_)) were performed according to the ATS/ERS guidelines using Vmax Sensor Medics [1]. Results were expressed as percentages of predicted values. PFTs were matched with HRCT examinations (mean distance between PFTs and HRCTs was 9.59 days ±44). All patients were treated, as recommended, with antifibrotic therapies (pirfenidone or nintedanib). No difference in terms of treatment dose was observed between deceased and non (*p* = 0.19).

### Quantitative analysis

HRCT examinations were acquired according to the following technical parameters: thickness ranged between 0.625–1.25 mm; sharp kernel imaging reconstruction, contiguous or overlap images; no contrast media administration. A user-friendly DICOM (digital imaging and communications in medicine) - based image processing software for automatic lung parenchyma segmentation was used to calculate HRCT quantitative parameters. This tool has been developed in the MATLAB R2016a simulation environment (The MathWorks, Natick, MA, USA), running on iMac (3,5 GHz Intel Core i7 processor, 16 GB memory random-access memory; Apple Computer, Cupertino, CA, USA) with Mac Operating System OS X El Capitan.

**Fig. 2:**
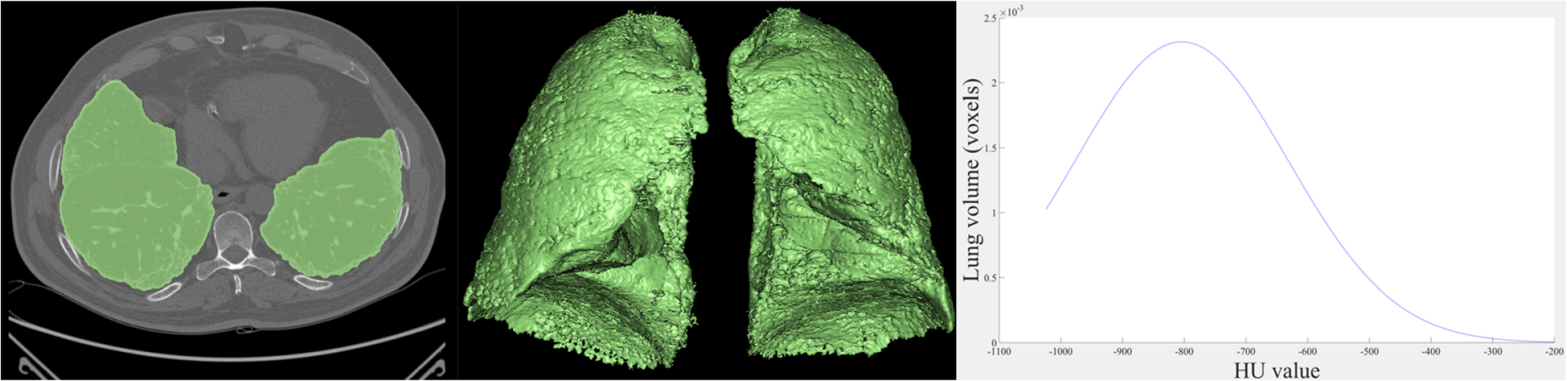
Example of lung parenchyma segmentation and CT-derived histogram

HRCT attenuation histogram was extracted after automatic lung parenchyma segmentation using region growing algorithm to include voxels between − 200 Hounsfield units (HU) and − 1.024 HU. kurtosis, Mean Lung Density (MLD), skewness, High Attenuation Areas (HAA) % and Fibrotic Areas (FA) % were subsequently calculated (Fig. 2).

It is known that kurtosis, skewness and MLD may provide prognostic estimation of disease severity in subjects with IPF and other ILDs. In particular, low kurtosis, low skewness and high MLD values may indicate severe IPF [8].

HAA may reflect the presence of parenchymal lesions, such as ground-glass opacity and reticulation. HAA% was calculated as percentages of the extracted whole lung volume with attenuation values ranging from − 250 HU to − 600 HU*.* In the same way, FA% was calculated considering values raging from − 1.024 HU to − 700 HU [9].

### Statistical analysis

Characteristics of the study population were expressed as mean (SD), median (interquartile range) or as percentage of the relative frequency as appropriated. Wilcoxon test for unpaired data was used to assess differences between baseline and follow up time. Reported data were adjusted using Holm’s correction. Spearman’s rank correlation was used to assess relation between CT-histogram based variables. Univariate and multivariate Cox proportional hazard regression analysis were performed to assess Hazard Ratios for predictors of survival. Akaike’s information criterion (AIC) was used to estimate the relative quality of the statistical models. Kaplan-Meier survival analysis was used to assess overall survival while ROC analysis was used to assess sensitivity and specificity. All the statistical analyses were performed using STATA/IC 14.2 version. A *p* less than 0.05 was considered significant.

## Results

The study evaluated 42 consecutive subjects with an MDT diagnosis of IPF. Thirty-five were male (83.33%) and 7 were female (16.67%). There were 33 (78.57%) former/current smokers and 9 (21.43%) never-smokers. The mean age at diagnosis was 68.26 ± 6.34 years. The mean follow up time was 1037 ± 430 days. Thirty-height (90.48%) patients were treated only with pirfenidone while 4 (9.52%) switched from pirfenidone to nintedanib because of drug-related adverse events. A total of 10 patients (23.33%) died during the follow up period. All demographic, PFTs and CT-derived variables at baseline are summarized in Table 1.

**Table 1:** Characteristics of study population

	Mean ± SD or n (%)	Median (range interquartile)
age at diagnosis (years)	68.26 ± 6.34	68 (64, 73)
male	35 (83.33)	
female	7 (16.67)	
current/former smoker	33 (78.57)	
never smoker	9 (21.43)	
follow up time (days)	1037.33 ± 430.04	978 (683.84, 1362)
FVC%^a^	84.28 ± 16.33	83.5 (74, 95)
DL_CO_%^b^	60.73 ± 17.16	61.5 (49, 69)
GAP (points)^c^	3.19 ± 1.10	3 (3, 4)
deceased	10 (23.81)	
pirfenidone only	38 (90.48)	
pirfenidone switched to nintedanib	4 (9.52)	
kurtosis	1.27 ± 1.07	1.05 (0.5, 1.77)
skewness	1.28 ± 0.33	1.29 (1.12, 1.51)
Mean Lung Density	− 770.77 ± 46.84	− 779.03 (− 795.90, − 755.98)
HAA^d^	15.31 ± 5.32	14.67 (11.91, 16.80)
Fibrotic Area%	25.78 ± 9.16	24.10 (19.55, 28.94)

^a^*FVC* Forced Vital Capacity, ^b^*DLCO* Diffusing Capacity for carbon monoxide, ^c^*GAP* Gender-Age-Physiology index, ^d^*HAA* High attenuation areas

A subset of 23 patients underwent a follow up HRCT within 12 ± 3 months from baseline. No significant differences were found in the variation of kurtosis, skewness, MLD, HAA%, FA%, FVC and GAP index (*p* = 0.25, *p* = 0.25, *p* = 0.40, *p* = 0.40, *p* = 0.68, *p* = 0.88, *p* = 0.21 respectively) (see Table 2).

**Table 2:** Differences between baseline and 12 ± 3 months

Variable	Baseline	12 ± 3 months	Interval difference	P^*^
kurtosis	1.26 (0.41, 2.26)	0.94 (0.01, 1.53)	−0.32	0.25
skewness	1.34 (1.07, 1.53)	1.15 (0.87, 1.41)	−0.19	0.25
MLD	− 777.92 (− 801.37, − 745.61)	− 764.76 (− 786.15, − 733.01)	−13.16	0.40
HAA%	14.42 (10.74, 17.79)	15.19 (12.81, 20.45)	0.77	0.40
FA%	24.68 (19.06, 32.00)	26.69 (22.14, 33.97)	2.01	0.68
FVC%	86 (75, 96)	85 (76.5, 105.25)	−1	0.88
DL_CO_%	67 (56, 72.50)	53 (40.75, 68.75)	−14	0.02
GAP (points)	3 (2.25, 4)	3 (3, 4)	0	0.21

Data are expressed as median (interquartile range)

^a^*MLD* Mean Lung Density, ^b^*HAA* High attenuation areas, ^c^*FA* Fibrotic Areas, ^d^*FVC* Forced Vital Capacity, ^e^*DLCO* Diffusing Capacity for carbon monoxide, ^f^*GAP* Gender-Age-Physiology index, * Reported *p*were adjusted with Holm’s correction

Univariate Cox proportional hazard regression analysis demonstrated, as expected, that both FVC% and DL_CO_% at baseline were significant predictors of survival (HR 0.93 and 0.93; *p* = 0.019 and *p* = 0.005). Similarly, also kurtosis, skewness, MLD, HAA% and FA% were good predictors of mortality (HR 0.44, 0.74, 1.01, 1.12, 1.06; *p* = 0.03, *p* = 0.01, *p* = 0.02, *p* = 0.02 and *p* = 0.017 respectively). Results of univariate Cox hazard regression are summarized in Table 3.

**Table 3:** Univariate Cox proportional hazard regression of the predictors of mortality

	HR	Std. Err^*^	P value	CI
kurtosis	0.44	0.16	0.03	0.209–0.924
skewness	0.74	0.075	0.01	0.010–0.537
MLD	1.01	0.006	0.02	1.002–1.025
HAA%	1.12	0.055	0.02	1.018–1.237
FA%	1.06	0.029	0.017	1.011–1.126
FVC%	0.93	0.024	0.019	0.892–0.989
DLCO%	0.93	0.022	0.005	0.893–0.980
Age	0.97	0.043	0.60	0.895–1.065
GAP (points)	1.92	0.64	0.052	0.994–3.724

^a^*MLD* Mean Lung Density, ^b^*HAA* High attenuation areas, ^c^*FA* Fibrotic Areas, ^d^*FVC* Forced Vital Capacity, ^e^*DLCO* Diffusing Capacity for carbon monoxide, ^f^*GAP* Gender-Age-Physiology index, *Std. Err Standard Error

Akaike’s information criterion (AIC) was calculated to estimate relative quality of the statistical model. Skewness demonstrated the lowest AIC value (AIC = 55.52) (see Table 4).

**Table 4:** Akaike’s information criterion for CT indexes

	kurtosis	skewness	MLD^b^	HAA%^c^	FA%^d^
AIC^a^	56.72	55.52	57.79	57.94	57.77

^a^*AIC* Akaike’s information criterion, ^b^*MLD* Mean Lung Density, ^c^*HAA* High attenuation areas, ^d^*FA* Fibrotic Areas

**Fig. 3:**
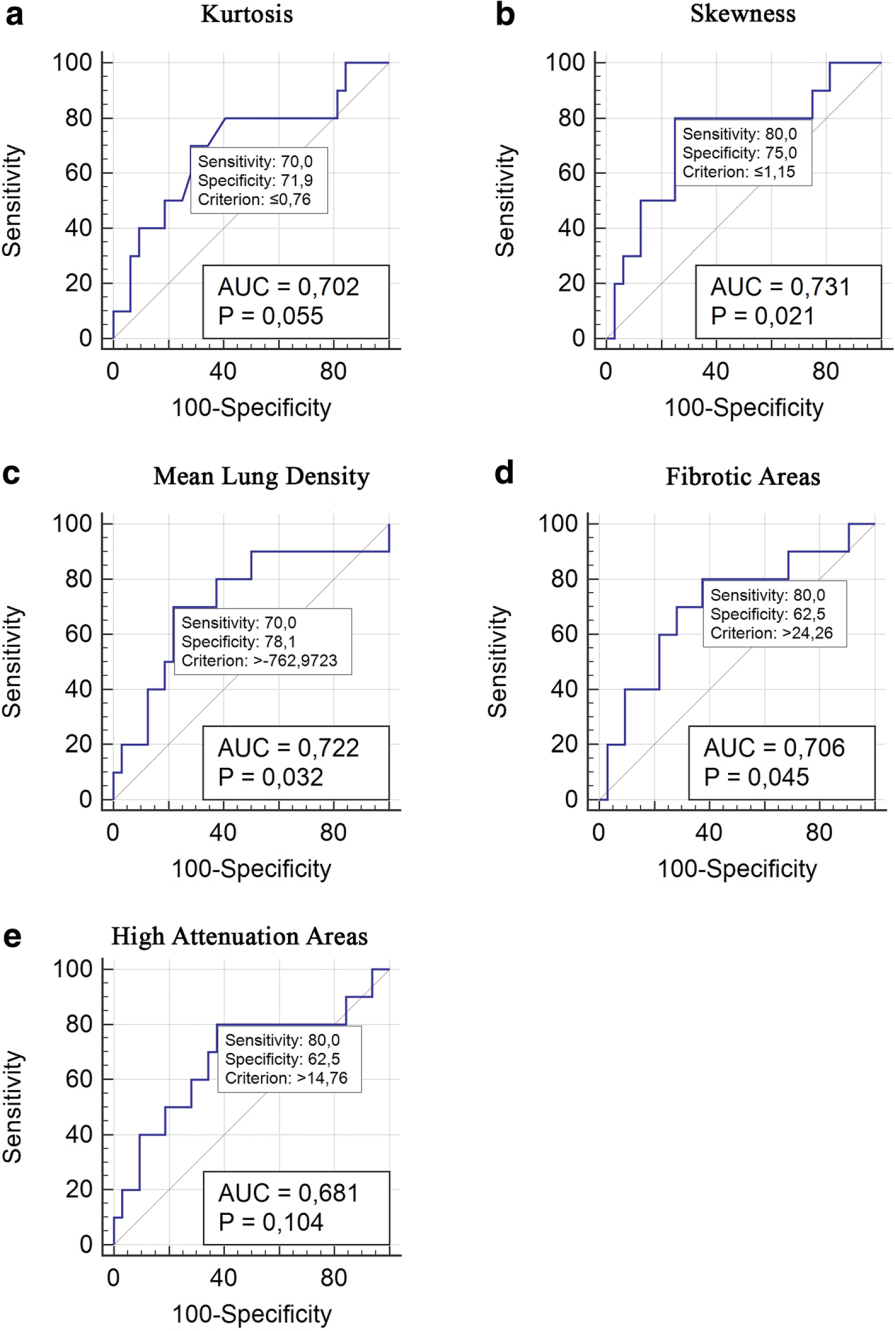
ROC analysis for kurtosis (**a**), skewness (**b**), MLD% (**c**), FA% (**d**), HAA (**e**)

A multivariate model was created considering FVC, DL_CO_, age and skewness, which was considered the best CT-derived variable because of the lowest AIC value. This model demonstrated an HR of 0.98, 0.94, 0.98 and 0.28 respectively for the four considered variables (AIC = 56.86) (see Table 5). ROC analysis shown in Fig. 3, demonstrated a sensitivity of 70% and a specificity of 71.9% for a threshold value of ≤0.76 of kurtosis (AUC = 0.70; *p* = 0.05), a sensitivity of 80% and a specificity of 75% for a threshold value of ≤1.15 of skewness (AUC = 0,731; *p* = 0.021), a sensitivity of 70% and a specificity of 78% for a threshold value of > − 762.97 of MLD (AUC = 0.722; *p* = 0.032), a sensitivity of 80% and a specificity of 62.5% for a threshold value of > 14.76 of HAA% (AUC = 0.681; *p* = 0.104) and a sensitivity of 80% and a specificity of 62.5% for a threshold value of > 24.26 of FA% (AUC = 0.706; *p* = 0.045) in predicting mortality.

**Table 5:** Multivariate Cox proportional hazard regression

	HR	Std. Err	P	CI
skewness	0.28	0.40	0.37	0.016–4.727
FVC^a^	0.98	0.03	0.59	0.919–1.049
DL_CO_^b^	0.94	0.02	0.06	0.897–1.003
Age	0.98	0.05	0.81	0.884–1.101

Akaike’s information criterion =56.86

^a^*FVC* Forced Vital Capacity, ^b^*DLCO* Diffusing Capacity for carbon monoxide

**Fig. 4:**
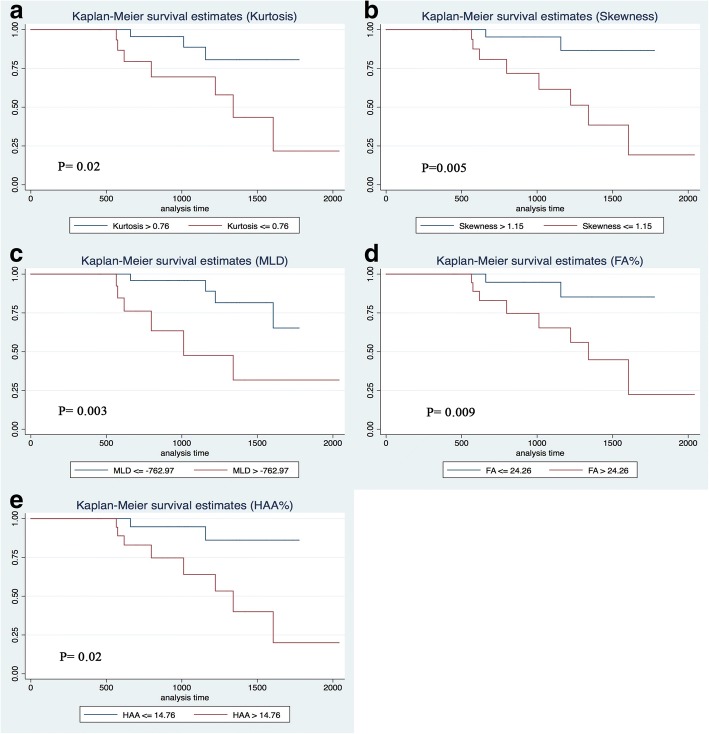
Kaplan-Meier curves for kurtosis (**a**), skewness (**b**), MLD (**c**), FA% (**d**), HAA% (**e**). Cut-off points: kurtosis ≤0.76; skewness ≤1.15; Mean lung density > − 762.97; FA% > 24.26; HAA% > 14.76

Each threshold value demonstrating the best sensitivity and specificity in predicting mortality was used as cut-off point to graph Kaplan-Meier curves specific for the CT indexes as shown in Fig. 4.

## Discussion

In recent years, HRCT has become a cornerstone in the diagnosis of IPF. Furthermore, the less-invasiveness of the exam compared to lung biopsy and the possibility of evaluating lung damage using quantitative analysis, has promoted HRCT as a reasonable marker of disease extension [10, 12–18]. To our knowledge, only three studies, evaluating the predictive value of CT histogram indexes in assessing survival, have been published [11, 19, 20].

Analysing these studies, we found some differences in terms of CT quantitative analysis protocols, treatments and duration of follow up. In particular, Tanizawa et al. and Ash et al. analysed scans (1 or 2 mm thick) every 10 mm of intervals while Best et al. analysed (1 mm thick) scans every 20 mm [19]. Best et al. considered patients enrolled in a double-blind placebo-controlled clinical trial of interferon β-1a while Tanizawa et al. evaluated a non-homogeneous cohort of IPF and other fibrotic ILDs that received multiple drugs (corticosteroids, immunosuppressive agents or pirfenidone) [11]. No information about treatment is reported in the study of Ash et al. [20]. These studies also differ regarding duration of follow up. Tanizawa et al. reported a median follow up time of 1,140 days while Best et al. and Ash et al. reported 547.5 and 465 days respectively.

For this reason, in our study, we tried to re-evaluate the predictive value of CT histogram indexes in assessing survival, analysing 1 mm thick scans without interval in a homogeneous cohort of consecutive IPF patients treated with antifibrotics and with a follow up time of 3 years.

Univariate Cox proportional analysis, as in previous studies, demonstrated that kurtosis, skewness, MLD, HAA% and FA% are significant predictors of mortality. Of all of these, skewness demonstrated the lowest AIC value and, according to our analysis, should be considered as the best CT histogram derived variable in predicting mortality. We also evaluated sensitivity and specificity of these variables with respect to mortality. Based on this, we identified possible cut-off points that we used to graph Kaplan-Meier curves. Although these cut-off points were extracted from a relatively small number of patients and need a validation in larger-group cohorts, we demonstrated how CT-variables and cut-off points may help in identifying patients with different life expectations. This finding could greatly help the clinician in the management of patients, especially if they are not able to correctly perform PFTs.

We also tested the role of skewness, FVC, DL_CO_ and age in predicting mortality in a Multivariate model. As shown in Table 5, although skewness is able to correctly predict mortality, it demonstrates in our model a less significant weight than DL_CO_, which was confirmed as a strong predictor of mortality.

Our study also evaluated changes after 12 ± 3 from baseline in a small subgroup of 23 patients. Differently from the study of Best et al., no difference was demonstrated, neither for CT quantitative variables nor for FVC changes. A possible explanation of this finding could reside in the treatment the patients received. In our study, subjects homogeneously received antifibrotic therapy resulting in a relative functional and radiological stability after one year. To our knowledge this represents the first study analysing patients treated with pirfenidone and nintedanib with this approach.

This study has some strengths. We analysed the entire volume of the lungs taking into account every thin section in the final calculation of the indexes; every exam was performed with the same protocol and in the same CT-scanner both at baseline and follow up; all patients were treated with antifibrotic drugs reproducing a real clinical setting; patients were followed for a long enough period to assess survival.

This study, however, also has several limitations. The retrospective design and the single-centre nature of the study represent the first great limitation. We also analysed a small number of patients, even if only the study of Best et al. considered a truly greater number of patients with this method. Our analysis considered HRCT scans performed with the same protocol and in the same CT-scanner. This is the reason for the exclusion of a large number of newly-diagnosed IPF patients over the considered period. Moreover, as is known, this kind of analysis may be greatly influenced by the level of inspiration of the patients. An incorrect manoeuvre or the presence of artifacts may erroneously affect quantitative CT indexes. Given the retrospective nature of the study, we did not used a spirometric control during acquisition to ensure an optimal inspiration. However, all CT-scans were reviewed and approved for the inclusion in the study by our radiologists. Another notable and previously mentioned limitation of the study includes the need for some computational steps required for the image processing to get the CT-indexes.

## Conclusions

Based on our results, baseline HRCT indexes are able to provide a prediction of survival and can be used as a surrogate marker for prognosis. Given the characteristics of simplicity, objectivity and reproducibility, this method could actually help the clinician and the radiologist in the evaluation of sub-clinical changes and the prognosis of patients in daily clinical practice. Further studies with larger cohorts and an external validation are needed to confirm these data.

## Abbreviations

AIC: Akaike’s information criterion
DLCO: Diffusing Capacity for carbon monoxide
FA: Fibrotic Areas
FVC: Forced vital capacity
GAP: Gender-Age-Physiology index
HAA: High attenuation areas
HRCT: High-resolution computed tomography
MLD: Mean Lung Density
